# AtWAKL10, a Cell Wall Associated Receptor-Like Kinase, Negatively Regulates Leaf Senescence in *Arabidopsis thaliana*

**DOI:** 10.3390/ijms22094885

**Published:** 2021-05-05

**Authors:** Lu Li, Kui Li, Akhtar Ali, Yongfeng Guo

**Affiliations:** 1 The Key Laboratory of Plant Development and Environmental Adaptation Biology, Ministry of Education, School of Life Sciences, Shandong University, Qingdao 266237, China; lu_li@sdu.edu.cn; 2 Tobacco Research Institute, Chinese Academy of Agricultural Sciences, Qingdao 266101, China; 968346616@126.com (K.L.); akhtar_arid@yahoo.com (A.A.)

**Keywords:** Arabidopsis, cell wall associated receptor-like kinase, AtWAKL10, leaf senescence, WRKY transcription factors, cell expansin, defense responsive

## Abstract

Receptor-like kinases (RLKs) constitute a large group of cell surface receptors that play crucial roles in multiple biological processes. However, the function of most RLKs in plants has not been extensively explored, and much less for the class of cell wall associated kinases (WAKs) and WAK-like kinases (WAKLs). In this study, analyses of developmental expression patterns uncovered a putative role of AtWAKL10 in modulating leaf senescence, which was further investigated at physiological and molecular levels. The expression level of *AtWAKL10* increased with the developmental progression and was rapidly upregulated in senescing leaf tissues. The promoter of *AtWAKL10* contains various defense and hormone responsive elements, and its expression could be significantly induced by exogenous ABA, JA and SA. Moreover, the loss-of-function *atwakl10* mutant showed earlier senescence along the course of natural development and accelerated leaf senescence under darkness and hormonal stresses, while plants overexpressing *AtWAKL10* showed an opposite trend. Additionally, some defense and senescence related WRKY transcription factors could bind to the promoter of *AtWAKL10*. In addition, deletion and overexpression of AtWAKL10 caused several specific transcriptional alterations, including genes involved in cell extension, cell wall modification, defense response and senescence related WRKYs, which may be implicated in regulatory mechanisms adopted by AtWAKL10 in controlling leaf senescence. Taken together, these results revealed that AtWAKL10 negatively regulated leaf senescence.

## 1. Introduction

Leaf development is a strictly programmed process that includes the early stages of leaf development such as initiation, growth, and maturation, and the later leaf senescence. Due to the sessile lifestyle of plants, leaf senescence is not only regulated by intrinsic and genetic factors, but also affected by various environmental stimuli, such as drought, shade, extreme temperature, and pathogen infections [1,2]. Leaf senescence is also a complicated multi-step process. In contrast to nutrient assimilation at early development stages, leaf senescence is mainly accompanied by highly ordered degradation of organelles and cellular compositions, subsequent remobilization of valuable resources from senescing leaves to vigorous tissues and organs, and eventually, the suspension of photosynthesis and programmed cell death [3,4,5]. Thus, following the initiation of leaf senescence, leaves will experience constant chlorophyll loss, reduced efficiency of photosynthesis, and increased accumulation of reactive oxygen species (ROS) [5,6,7]. To cope with the substantial metabolic loss during leaf senescence, plants have established sophisticated transcriptional regulatory networks and signaling mechanisms to support plant growth and ensure reproduction by stimulating the expression of senescence associated genes (*SAGs*) [2,8,9].

The initiation of natural leaf senescence is mainly dependent on the age [10], but can also be triggered by various exogenous stimuli. As crucial tissues for photosynthesis, leaves subjected to prolonged light deprivation such as complete darkness show accelerated yellowing, which also exhibit a considerable overlap in differentially expressed genes and signaling pathways with natural senescence [11]. However, it is worth noting that such senescence phenotypes are more suitable for individual and detached leaves but not the whole plant [12,13]. In addition to dark treatment, plant hormones are also commonly used as effective inducers of senescence. Hormone signaling pathways, as a comprehensive mechanism, are involved in regulating both developmental and environmental responses. Many studies have found that the levels of hormones such as abscisic acid (ABA), jasmonic acid (JA) and salicylic acid (SA) are increased in senescing leaves [14,15,16], which may promote senescence by inducing *SAGs* [17,18]. Correspondingly, the transcription levels of genes encoding key components for hormonal biosynthesis and signal transduction are also altered during leaf senescence [16,19,20]. 

During leaf senescence, genes involved in catabolism and defense responses undergo large-scale transcriptional reprogramming, which requires hierarchical but also coordinated regulation by multiple transcription factors (TFs) [11,16,21]. It is well exemplified by the regulation of chlorophyll degradation. Specifically, chlorophyll catabolic genes such as *NON-YELLOWING1* (*NYE1*)/*STAY-GREEN1* (*SGR1*) and *PHEOPHORBIDE A OXYGENASE* (*PAO*) can be induced by increased ABA accumulation [22]. The ABA biosynthesis gene *ALDEHYDE OXIDASE3* (*AAO3*) is a transcription target of NAC-LIKE, ACTIVATED BY AP3/PI (NAP/ANAC029) in Arabidopsis, while the promoter of *AtNAP* can be bound by ANAC016, which is also a transcription regulator of *NYE1* [23,24]. Moreover, *NYE1*, and *PAO* are positively regulated by MYC2, MYC3, and MYC4, three positive TFs of the JA signaling pathway [25], while they are also integrated into the regulatory loop of ethylene-induced chlorophyll degradation with ETHYLENE-INSENSITIVE3 (EIN3) and ORESARA 1 (ORE1)/ANAC092 [26]. In addition to NACs, WRKY TFs also constitute a large set of *SAGs*, some of which also play a role in other biological processes, typically, hormone mediated defense responses [11]. For example, *WRKY6* is upregulated upon pathogen infection and leaf senescence [27,28], and overexpression or lack of WRKY6 facilitates and retards dark-induced senescence [29]. It is also required for SA-mediated leaf senescence, and transcriptionally activated by WRKY46 upon SA treatment [30]. Both WRKY6 and WRKY46 are downstream of NONEXPRESSER OF PR GENES 1 (NRP1), and act synergistically with NRP1 to positively regulate leaf senescence. 

Plant receptor-like kinases (RLKs) are the largest serine/threonine protein kinase subfamily, which can mediate extracellular signals and then activate intracellular signaling pathways, thus playing a crucial role in cell-cell communications and plant-pathogen interactions [31,32,33]. Wall associated kinases (WAKs) and WAK-like kinases (WAKLs) are a small subset of RLKs with a total of 26 members in Arabidopsis, which possess a unique extracellular region that harbors several epidermal growth factor (EGF) repeats and is closely associated with the cell wall [34,35]. In addition, the composition of the extracellular domain of each member is also different, which may be due to their different ligand types and regulatory functions [35,36]. WAKs are essential for cell expansion, and some members, such as WAK1, are primarily involved in defense responses and can be up-regulated by fungal pathogens, exogenous SA, MeJA, and systemically acquired resistance (SAR) [37,38,39,40]. AtWAKL10 has similar molecular weight and structural characteristics to WAK1 and has been identified as a twin-domain signaling molecule [41,42]. Its intracellular domain has both kinase activity and functional Guanylyl cyclase (GC) activity, which can catalyze the formation of guanine nucleoside 3’,5’-cyclomonophosphate (cGMP), an important signaling molecule involved in a variety of physiological and biological processes in higher plants [41]. A more recent study also showed that *AtWAKL10* could be induced by nitric oxide (NO) and played a positive role in basal defense, effector-triggered immunity and salt stress but negatively regulated drought stress [43].

Leaf senescence is a natural and orderly physiological process, which is of great significance to agricultural production. Under unfavorable conditions, the timely initiation of senescence is beneficial to plant viability and successful regeneration. Here, based on tissue expression pattern analyses on a variety of RLKs, *AtWAKL10* was found to be upregulated in senescent leaves. This was further confirmed by quantification of its transcript abundance in leaf tissue of different degrees of senescence. Characterization of the loss-of-function *atwakl10* mutant and plants overexpressing AtWAKL10 found that they were sensitive and resistant to natural, dark-, and hormone-induced leaf senescence, respectively. Moreover, several defense and senescence associated WRKYs could bind to the promoter of *AtWAKL10*, while expression of some WRKYs could also be affected by the levels of AtWAKL10, further indicating that AtWAKL10 was involved in regulating leaf senescence. In addition, deletion and overexpression of AtWAKL10 caused significant changes in expression of cell expansins and defense response genes, respectively, which only occurred during leaf senescence. These results indicated that AtWAKL10 played a negative role in leaf senescence. 

## 2. Results

### 2.1. Functional Domain Analysis and Subcellular Localization of AtWAKL10

**Figure 1:**
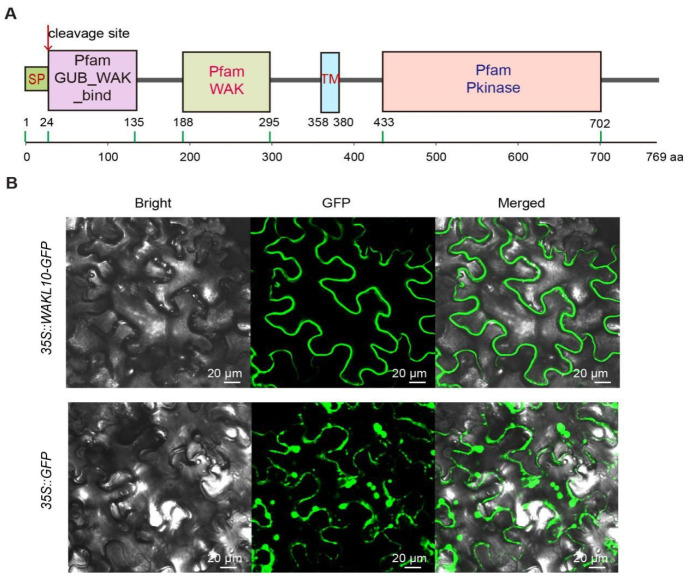
The domain composition and subcellular localization of AtWAKL10. (**A**) Diagram showing the significant domains and motifs predicted by the SMART program (http://smart.embl.de/, accessed on 12 March 2020). A signal peptide (SP) of 24 aa (aa 1 to 24) predicted by the SignalP-5.0 program (http://www.cbs.dtu.dk/services/SignalP/, accessed on 19 April 2021) is indicated in the light green box and the cleavage site is indicated with a red arrow. The accession for GUB_WAK, WAK, and protein kinase (Pkinase) is PF13947, PF08488, and PS5001, respectively. The amino acid (aa) positions for each domain are indicated by numbers above the green lines. TM: transmembrane domain. (**B**) *35::AtWAKL10-GFP* and *35::GFP* constructs were transiently transformed and expressed in *Nicotiana benthamiana* leaf epidermal cells for 3 d, then GFP signals were visualized on a confocal laser microscope.

Functional domains along the AtWAKL10 protein sequence were identified through the SMART v9 service program and a signal peptide of 24 aa (aa 1 to 24) with a likelihood of 0.991 as well as the corresponding cleavage site at Ala24-Ser25 were predicted by using the SignalP-5.0 program (Figure 1A). AtWAKL10 is a typical wall-associated kinases like transmembrane protein that has a cytoplasmic serine/threonine kinase domain (aa 433 to 702) and an extracellular cysteine-rich galacturonan-binding (GUB_WAK_bind) domain (Figure 1A), which functions in cellular activity by phosphorylation and associating cell membrane to cell-wall pectin, respectively [34,41,44,45]. The WAK kinase domain (aa 188 to 295) is required for cell expansion and involved in signaling to the cytoplasm [36].

To determine the subcellular localization of AtWAKL10, the full-length CDS sequence without stop codon was cloned and fused to the N-terminal of GFP in the pCHF3-CGFP vector. Then, the resulting *35S::AtWAKL10-GFP* fusion construct and *35S::GFP* were transiently transformed in *N. benthamiana* leaf epidermal cells, respectively. Unlike the random distribution of signals of *35S::GFP* in the cytoplasm, *AtWAKL10-GFP* seemed to be localized to cell membranes (Figure 1B).

### 2.2. AtWAKL10 Was Highly Induced during Leaf Senescence

To identify putative roles of AtWAKL10 in plant development, tissue expression pattern of *AtWAKL10* was analyzed through Arabidposis eFB Brower (http://bar.utoronto.ca, accessed on 8 November 2020) and GENEVESTIGATOR v3 (https://genevestigator.com, accessed on 20 December 2020). *AtWAKL10* showed relatively low expression in most plant tissues and developmental stages but was clearly upregulated in senescing leaves (Supplementary Figure S1). 

**Figure 2:**
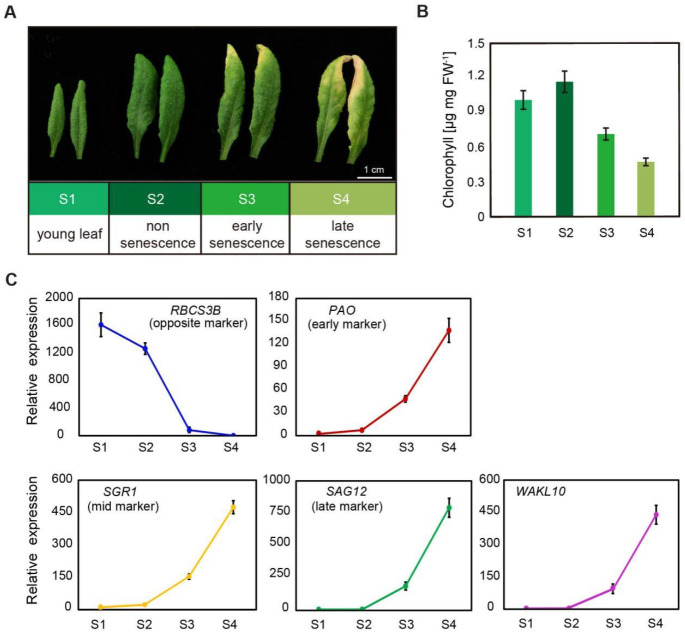
The specifically upregulated expression of *AtWAKL10* in leaves of *A. thaliana* wild type (Col-0) plants during natural senescence. (**A**) Representative images of fifth and sixth rosette leaves from *A. thaliana* wild type (Col-0) plants at different developmental stages. S1, young leaf; S2, non-senescence leaves with true leaves fully expanded; S3, early senescent leaf with yellowing leaf area less than 25% from the leaf tip; S4, more than 40% of leaf area yellowing. (**B**) The chlorophyll concentrations in leaves 5 and 6 at S1, S2, S3, and S4 stages. (**C**) The transcript abundance of *AtWAKL10* and leaf senescence marker genes. Data were given as averages ± SE of three biological replicates. For *RBCS3B*, gene expression was calculated relative to S4 stage, or otherwise, was relative to S1 stage.

To confirm the expression pattern, rosette leaves 5 and 6 from *A. thaliana* wild type (Col-0) plants at four different developmental stages were collected, including young leaf stage (S1), non-senescence mature leaf stage (S2), early senescence stage (S3), and late senescence stage (S4) (Figure 2A). A constant decrease in chlorophyll concentrations in leaves from S1 to S4 indicated the progression of leaf senescence (Figure 2B). This was further supported by the expression pattern of typical senescence marker genes (Figure 2C). The expression levels of senescence marker genes *PAO* and *NYE1*/*SGR1* increased slightly during the transition from young leaves to mature leaves, and then increased continuously and rapidly with the developmental stages (Figure 2C). Correspondingly, the senescence-opposite maker gene, the small subunit of *1,5-ribulose bisphosphate carboxylase oxygenase* (*RBCS3B*) consistently declined (Figure 2C). A sharp decrease in its expression from S2 to S3 stage also indicated the early onset of leaf senescence. It was not surprising that the age-related senescence marker gene *SAG12* was specifically and substantially upregulated in leaves at both early and late senescence stages (Figure 2C). For the *AtWAKL10* transcript, it showed highly consistent expression patterns with *SAG12*, which was rapidly induced from the initial leaf senescence (Figure 2C), suggesting that *AtWAKL10* may be involved in the regulation of leaf senescence.

### 2.3. The Expression Patterns of AtWAKL10 in Various Leaf Tissues

**Figure 3:**
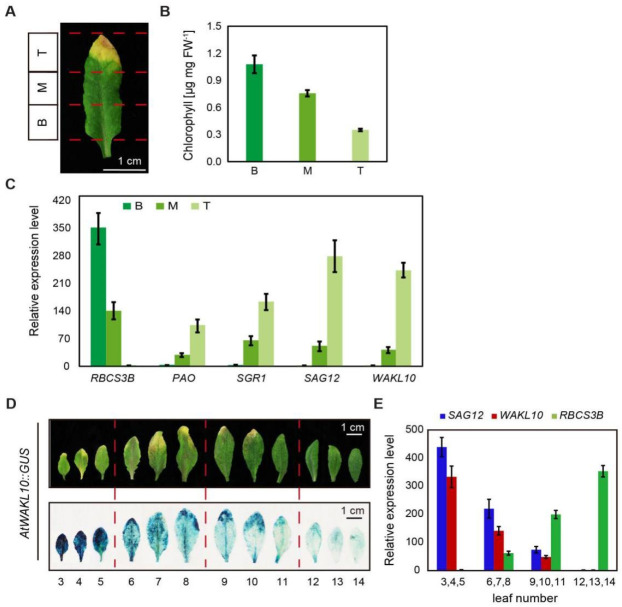
The expression levels of *AtWAKL10* in various leaf tissues. (**A**) Representative image of an individual rosette leaf 7 from 8-week-old *A. thaliana* wild type (Col-0) plants. (**B**) Chlorophyll concentrations, and (**C**) transcript abundance of *AtWAKL10* and senescence related marker genes in three leaf sections: T, leaf tip; M, middle part; and B, leaf base. (**D**) GUS staining on rosette leaves from a whole plant of *AtWAKL10::GUS* transgenic line. The rosette leaves were divided into three groups according to the GUS intensity. (**E**) The transcript levels of *AtWAKL10* and two senescence marker genes in each group. The relative expression levels of all tested genes except *RBCS3B* were calculated relative to the least senescent section (leaf base) or group (leaves 12, 13 and 14). *RBCS3B* was calculated in an opposite way. Data were given as averages ± SE of three biological replicates.

To further confirm the up-regulated expression of *AtWAKL10* in senescing leaves, the transcript abundance of *AtWAKL10* in different parts of senescing leaves was evaluated. It is well known that the natural senescence of a single leaf extends from leaf tip to the base, so the true leaf 7 from 8-week-old *A. thaliana* wild type (Col-0) plants was divided into three parts, namely, leaf tip, middle leaf, and leaf base (Figure 3A). As expected, chlorophyll concentrations decreased from the base section to the leaf tip (Figure 3B), and the lowest transcript abundance of *RBCS3B* and the highest for *SAG12* were found in the tip section (Figure 3C). The expression patterns of *AtWAKL10* were similar to that of *SAG12*, which increased continuously from leaf base and was highly upregulated in senescent leaf tips (Figure 3C). In addition, senescence-dependent up-regulation of *AtWAKL10* was further checked using transgenic GUS plants driven by the *AtWAKL10* promoter, a ~2.3 kb fragment upstream of the start codon. The results showed that almost no GUS signals were observed in young leaves, while there was an increasing accumulation of GUS signals as leaf ageing progressed (Figure 3D). Then, rosette leaves were divided into four groups according to GUS signal intensity, and the senescence degree of leaves in each group was represented by the transcription levels of *SAG12* and *RBCS3B* (Figure 3E). Consistent with GUS signal intensity, the expression of *AtWAKL10* was the highest in the oldest leaves 3, 4, and 5, followed by the sub-senescent leaves 6, 7, and 8, and lowest in the young leaves 12, 13, and 14 (Figure 3E). Together, these results confirmed that *AtWAKL10* was highly induced by natural senescence.

### 2.4. AtWAKL10 Negatively Regulates Natural Leaf Senescence

**Figure 4:**
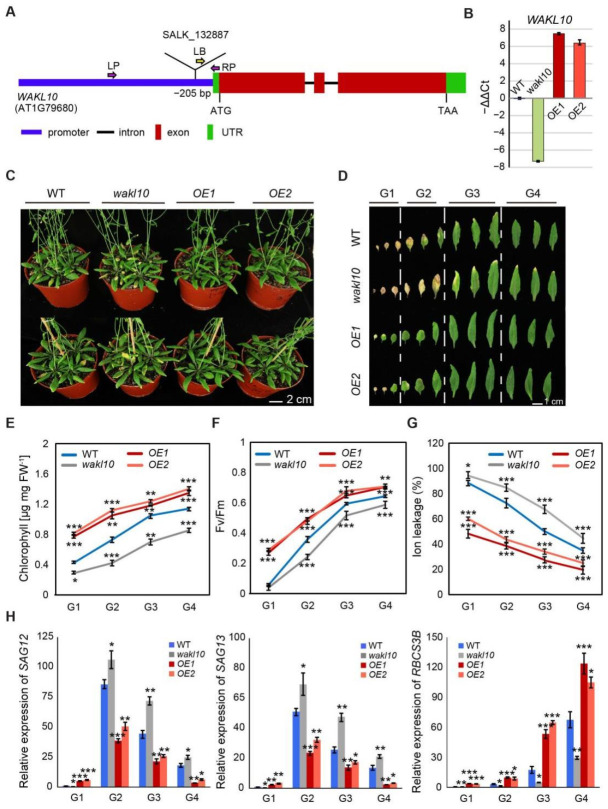
AtWAKL10 negatively regulated natural leaf senescence. (**A**) Schematic gene model of *AtWAKL10*. The T-DNA insertion site was shown, and homozygous mutants were screened using specific primers (Supplementary Table S1). LP (left primer), RP (right primer), left border primer (LB), ATG (start codon), TAA (stop codon) and UTR (untranslated region). (**B**) Quantification of *AtWAKL10* in *atwakl10* mutant and *AtWAKL10* overexpression lines. Expression levels of *AtWAKL10* were determined by qRT-PCR and presented as values of −ΔΔCt. Data shown were means ± SE of three independents plants. (**C**) Phenotypes of 7-week-old wild type (Col-0), *atwakl10* mutant and *AtWAKL10* overexpression lines (*OE1* and *OE2*), and (**D**) a series of developmental stages of all leaves as indicated. Rosette leaves were divided into four groups according to the degree of senescence. Quantification of chlorophyll concentrations (**E**), PSII maximum efficiency (Fv/Fm) (**F**), ion leakage (**G**) and relative expression of *SAG12, SAG13* and *RBCS3B* (**H**) in leaves from each group. Mean values ± SE were shown for three biological replicates. Significant differences (* *p* < 0.05, ** *p* < 0.01, and *** *p* < 0.001) compared with the wild type in each group were determined by Student’s *t*-test.

To investigate how AtWAKL10 functions in regulating natural leaf senescence, a homozygous mutant line designated as *atwakl10* (SALK_132887) was screened by PCR (Supplementary Figure S2). Sanger sequencing revealed that the mutant contained a T-DNA insertion in the promoter region, specifically, 205 bp upstream of the start codon (Figure 4A, Supplementary Figure S2). Meanwhile, two overexpression lines for AtWAKL10 were generated in the *A. thaliana* wild type (Col-0) background, designated as *AtWAKL10 OE1* and *AtWAKL10 OE2* (Figure 4A). Both the knock-out mutant and overexpression lines were confirmed by quantitative Real-time PCR (Figure 4B). 

Soil-based phenotyping was performed on the wild type, *atwakl10* mutant, and *AtWAKL10 OE* lines, and no obvious differences in leaf size, leaf number, and flowering time were observed. Then, a closer analysis of developmental progression revealed that natural leaf senescence seemed to occur earlier for the loss-of-function *atwakl10* mutant, while overexpression of AtWAKL10 greatly inhibited leaf senescence (Figure 4C). Specifically, compared with the wild type, the 7-week-old *atwakl10* mutant had about two more yellowing leaves and a greater degree of chlorophyll loss in the same leaf position, while more than 90% of the true leaves from the *AtWAKL10 OE* plants remained green (Figure 4D). Again, the rosette leaves from a single plant were divided into four groups based on the leaf age. In agreement with the severe leaf senescence phenotype, leaves of each group from *atwakl10* mutant had the lowest chlorophyll concentrations and quantum efficiency of photosystem II (Fv/Fm) (Figure 4E,F). Meanwhile, it was also supported by the higher expression of *SAG12* and lower expression of *RBCS3B* in almost all groups. One exception for the lower expression of *SAG12* and *SAG113* in the oldest leaves (group1) was due to the loss of leaf vitality [46]. On the other hand, not surprisingly, the values for various physiological parameters and the transcription abundance of senescence marker genes in all groups of *AtWAKL10 OE* plants were significantly greater than that in wild type (Figure 4E,F,H). In addition, with the course of leaf senescence, ion leakage increases due to disruption of cell membranes and increased membrane permeability [47]. As was shown, the accelerated senescence symptoms of the *atwakl10* mutant were accompanied by the higher membrane ion leakage rates (Figure 4G), whereas two overexpression lines always had lower ion leakage rates compared with the wild type. These results further suggested that AtWAKL10 exerted negative control on natural leaf senescence. 

### 2.5. AtWAKL10 Had a Negative Effect on Dark-Induced Senescence

**Figure 5:**
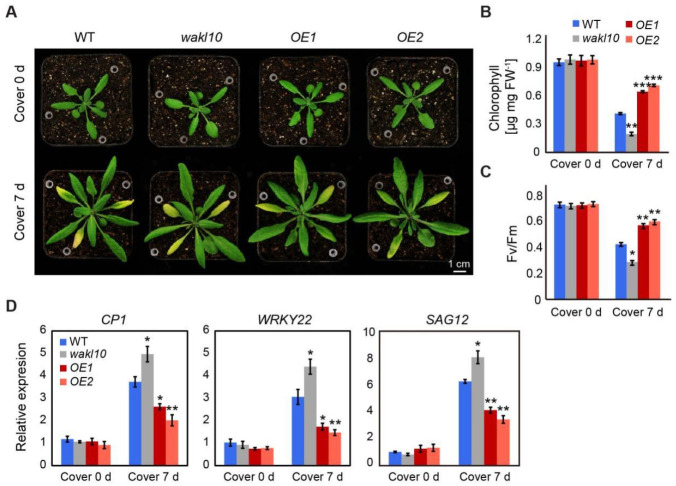
AtWAKL10 effectively delayed dark-induced senescence in attached leaves. (**A**) Phenotypes of rosette leaves from *A. thaliana* wild type (Col-0), *atwakl10* mutant, and *AtWAKL10* overexpression lines under control growth conditions and after 7 d of darkness treatment. The 25-day-old uniformly grown plants were designated as cover 0 d, and those subjected to a follow-up extra 7 d of dark acclimation were indicated as cover 7 d. Attached leaves 4, 5, and 6 were exposed to darkness stress by being covered with aluminum foil paper. The tested leaves were indicated by white pipette tips. Quantification of chlorophyll concentrations (**B**), chlorophyll fluorescence efficiency (Fv/Fm) (**C**), and expression levels of dark-induced or natural senescence related genes (**D**) in tested leaves before and after 7 d of darkness stress. Data were given as averages ± SE of five biological replicates. Significant differences compared with wild type under each condition were indicated (* *p* < 0.05, ** *p* < 0.01, and *** *p* < 0.001, Student’s *t*-test).

To further elucidate the roles of AtWAKL10 in leaf senescence, darkness stress was applied to induce leaf senescence. To start, dark-induced senescence was performed on attached leaves 4, 5, and 6 from 25-day-old wild type, *atwakl10* mutant, and *AtWAKL10 OE* plants by covering leaves with aluminum foil paper for 7 d (Figure 5A). Although no differences were observed in chlorophyll concentrations and photosynthetic efficiency (Fv/Fm) among these genotypes under control conditions, the values of these parameters were significantly lower in *atwakl10* mutant but higher in both *AtWAKL10 OE* lines compared with wild type after 7 d of dark treatment (Figure 5B,C). In addition, three senescence associated marker genes, including *WRKY22*, *CYSTEINE PROTEASE 1* (*CP1*), and *SAG12*, were upregulated in all genotypes after 7 d of darkness stress, and the corresponding expression levels were always the highest in the *atwakl10* mutant but the lowest in both *AtWAKL10 OE* lines.

**Figure 6:**
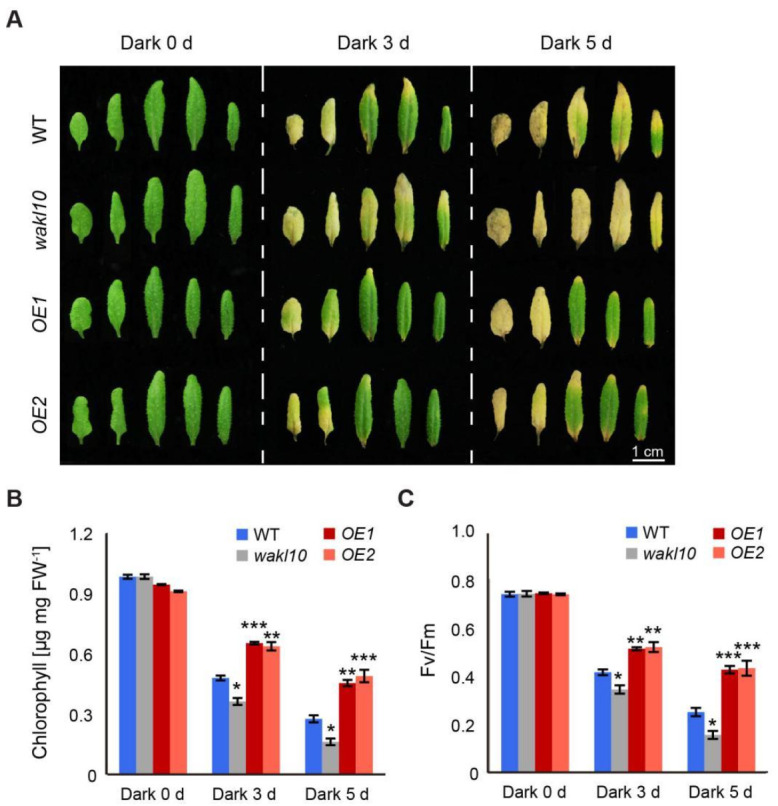
AtWAKL10 delayed dark-induced senescence in detached leaves. (**A**) Representative images of detached leaves 3, 4, 5, 6, and 7 from 25-day-old plants of tested genotypes before and after a 3 d and 5 d of darkness acclimation. Leaves were placed on moistened filter papers in foil-wrapped Petri dishes and collected at indicated time points for measurements of chlorophyll concentrations (**B**) and photosynthetic efficiency (Fv/Fm) (**C**). Error bars showed the SE (*n* = 5). Significant differences compared with the wild type at each time point were determined by a Student’s *t*-test (* *p* < 0.05, ** *p* < 0.01, and *** *p* < 0.001). Three independent experiments were carried out with similar results.

Furthermore, dark-induced senescence was also carried out on detached leaves. Individual leaves 3, 4, 5, 6, and 7 from 25-day-old plants were excised and placed on moistened filter paper, which was covered with aluminum foil paper to induce senescence. After 3 d of dark treatment, all leaves from the *atwakl10* mutant experienced substantial loss in chlorophyll content by a total of more than 60%, while it was reduced by approximately 48% in the wild type (Figure 6A,B). By contrast, relatively less chlorophyll breakdown occurred in the third and fourth leaves of both *AtWAKL10 OE* lines, with the remaining leaves staying green (Figure 6A). Furthermore, following 5 d of acclimation to darkness, the difference in senescence severity of tested genotypes was more pronounced. Specifically, the chlorophyll concentration in the wild-type and *AtWAKL10 OE* plants was ~1.8 times and 2.7 times higher than that of the mutant, respectively (Figure 6B). The changes in photosynthetic efficiency also showed a similar trend (Figure 6C). Overall, these results suggested that AtWAKL10 negatively regulated dark-induced senescence.

### 2.6. Promoter Analysis of AtWAKL10

**Figure 7:**
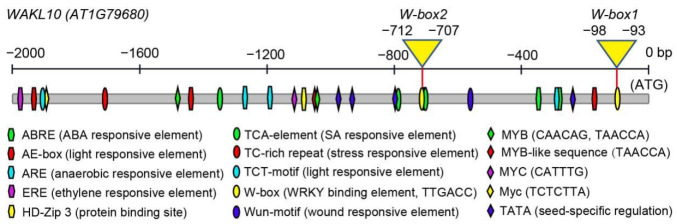
Analysis of the promoter of *AtWAKL10*. Schematic diagram of the promoter region of *AtWAKL10* showing various stress responsive cis elements and transcription factors binding sites. The positions of two W-box motifs are indicated by yellow solid triangles.

To gain better insights into the potential upstream transcription regulators of *AtWAKL10*, the 2000 bp promoter region sequence was obtained from the Arabidopsis Information Resource TAIR 10 (https://www.arabidopsis.org/, accessed on 23 December 2020) and analyzed through the database search program PlantCARE. A diversity of cis elements associated with hormone responses, stress responses, development, and light response were predicted (Figure 7A). Among these, hormone response elements were abundant in the *AtWAKL10* promoter region, including two ABA-responsive elements (ABREs), one ERE-element (ethylene response element), and three TCA elements (salicylic acid response element). Stress responsive elements such as ARE (anaerobic responsive element) and Wun-motif (wound responsive element) were presented in the promoter region. In addition, two types of elements related to light response (AE-box and TCT-motif) and TATA element involved in regulation of seed development were identified. More importantly, the *AtWAKL10* promoter also contained various transcription factor binding sites (TFBS), including three MYB binding sites, two MYC binding sites, and two WRKY binding sites (W-box). The dense distribution of hormone-responsive elements, stress-responsive elements, and typical TFBS strongly suggested that *AtWAKL0* may be involved in a variety of hormone or pathogen-defense-related regulatory pathways, which had been partially confirmed by previous studies [41,43]. 

### 2.7. The Expression Pattern of AtWAKL10 in Response to Various Hormones

**Figure 8:**
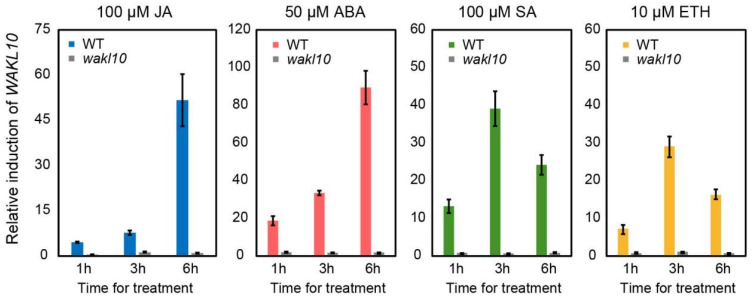
The expression pattern of *AtWAKL10* in response to various hormones. Ten-day-old seedlings of wild type and *atwakl10* mutant were water cultured on 1/2 MS medium supplemented with 100 µM JA, 50 µM ABA, 100 µM SA, and 10 µM ETH for 6 h, respectively. Samples were collected at different time points as indicated. For each genotype, the relative induction in abundance of *AtWAKL10* under each treatment condition was calculated relative to the mock-treated expression levels after being normalized against *ACT2*.

The promoter region of *AtWAKL10* was predicted to contain a variety of hormone responsive elements, such as SA, ABA, and ethylene. Previous studies have shown that *AtWAKL10* could be induced by SA [41]. Here, we further investigated its response to other hormones (Figure 8). It was clear that *AtWAKL10* was upregulated under all treatments, among which ABA treatment caused the highest relative induction, followed by JA treatment, and ethephon (ETH) treatment was the lowest (Figure 8). As expected, no induction in expression of *AtWAKL10* was detected in the knock-out *atwakl10* mutant under all stresses. The patterns of relative induction in the *AtWAKL10* transcript varied with hormone stress. Specifically, a similar trend with a continuous increase was observed under JA and ABA treatments (Figure 8A,B). In contrast, it increased sharply from 1 h, peaked at 3 h, then decreased at 6 h upon SA treatment (Figure 8C). The results indicated that AtWAKL10 had an early response to SA, which was in agreement with a previous study [41]. A similar trend was observed for ETH treatment, but to a lesser extent (Figure 8D). 

### 2.8. AtWAKL10 Negatively Regulates Hormone-Induced Senescence

A growing body of evidence suggests that hormones such as ABA, JA, and SA can promote leaf senescence. Since AtWAKL10 was found to play a regulatory role in leaf senescence and respond to hormone treatments, we intended to further explore its role in hormone-induced leaf senescence. 

**Figure 9:**
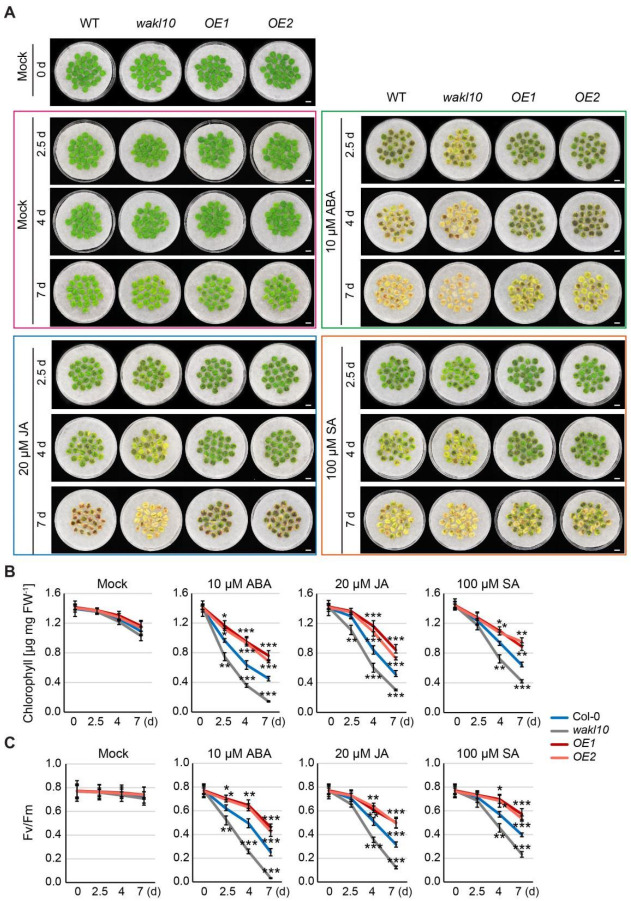
AtWAKL10 negatively regulated hormone mediated senescence in Arabidopsis. (**A**) Hormone responsive phenotypes of leaf discs with equal area (diameter = 1 cm) taken from leaves 7 and 8 of 4-week-old wild type, *atwakl10* mutant, and *AtWAKL10 OE* plants. More than 20 leaf discs per genotype were untreated (Mock) and treated with various hormones as indicated. Representative images of leaf discs with obvious senescence phenotypes were taken before hormone treatment and after 2.5 days, 4 days, and 7 days of mock treatment (purple box), 10 μM ABA treatment (green box), 20 μM JA treatment (light blue box), and 100 μM SA treatment (orange box) as indicated, respectively. Mock treated leaf discs were used to monitor natural senescence progression. All scale bars indicate 1 cm. (**B**) The chlorophyll concentrations and (**C**) Fv/Fm in hormone treated and untreated leaf discs as indicated in (**A**). Data were presented as means ± SE (*n* > 20). For all conditions, statistically significant differences compared with the wild type at each time point were determined by Student’s *t*-test (* *p* < 0.05; ** *p* < 0.01 and *** *p* < 0.001). All treatments were performed for three times.

Phenotypic analysis of hormone-induced senescence was performed on leaf discs isolated from leaves 7 and 8 of wild type (Col-0), *atwakl10* mutant, and *AtWAKL10 OE* plants. As expected, chlorophyll in non-senescent leaf discs from all tested 4-week-old genotypes did not show drastic decomposition under mock treatment (Figure 9A), whereas when using 6-week-old plants that started to initiate natural senescence, there was significant continuous chlorophyll degradation following 5 d of mock treatment (Supplementary Figure S3). The degradation of chlorophyll in *atwakl10* mutant was accelerated, which was consistent with its premature leaf senescence phenotype during natural development progression (Figure 4C,D). Contrary to the mutant, chlorophyll breakdown in both overexpression lines was slower than that in the wild type (Supplementary Figure S3). These results further supported the negative regulation of natural leaf senescence by AtWAKL10.

As ABA, JA, and SA can trigger leaf senescence, it was not surprising that hormone-treated leaf discs had significantly lower chlorophyll concentrations and PSII maximum efficiency (Fv/Fm) compared with the corresponding mock-treated leaf tissues at all time points of treatment (Figure 9, Supplementary Figure S3). Additionally, it could be seen that the promoting effect on leaf senescence varied with the types of hormones. Specifically, a rapid initiation of leaf senescence in 2 days was mediated by ABA while visible leaf yellowing phenotypes were observed after 4 d of treatment by SA or JA (Figure 9A). A similar trend in responding to different hormones was also observed in leaf discs from older plants (Supplementary Figure S3). Once in the presence of ABA, JA, or SA, both *AtWAKL10 OE* lines showed higher chlorophyll content and Fv/Fm than the wild type while the *atwakl10* mutant had the lower (Figure 9B,C). More specifically, compared to the corresponding control conditions, the relative reductions in chlorophyll concentrations and photosynthetic efficiency following each hormone stress were greater in *atwakl10* mutant and less in *AtWAKL10 OE* lines compared with the wild type. Altogether, these results further indicated that AtWAKL10 had an inhibitory effect on natural senescence as well as ABA-, JA-, and SA-induced leaf senescence. 

### 2.9. Identification of Defense and Senescence Related WRKYs Binding to the Promoter of AtWAKL10

**Figure 10:**
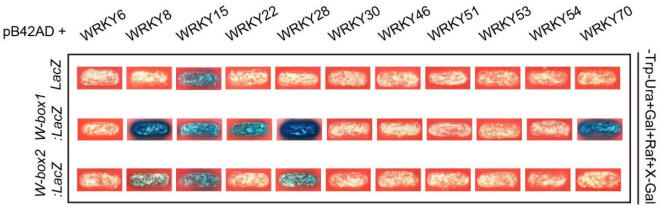
Identification of defense and senescence related WRKYs binding to the promoter of *AtWAKL10*. Yeast one-hybrid assays indicated that most WRKYs bound to the promoter region of *AtWAKL10* through the W-box motif nearby the start codon. Various WRKY transcription factors involved in defense response or leaf senescence were cloned into the *pB42AD* vector. Two fragments containing the W-box motif as indicated were inserted into the *pLacZi* vector, respectively. EGY48 yeast stains carrying two vectors were grown on SD/-Trp-Ura+Gal+Raf+X-Gal medium. Colonies in blue color indicate the positive interaction between protein and DNA motif.

WRKY transcription factors have been found to play important regulatory roles in defense response and natural or dark-induced leaf senescence. Promoter analysis of *AtWAKL10* revealed that it contained two core WRKY binding motifs at −93 nucleotides and 707 nucleotides upstream of the start codon, namely W-box1 and W-box2 (Figure 7). Additionally, a set of WRKYs have been predicted to bind the promoter region of *AtWAKL10* [41,43]. Here, we specifically focused on the binding of the *AtWAKL10* promoter by defense and senescence associated WRKYs. A total of ten reported WRKYs, including WRKY6/8/15/22/28/30/46/51/53/54/70, were cloned into the pB42AD vector (Figure 10). A promoter fragment harboring W-box1 or W-box2 motif was inserted into the *pLaczi* vector. Each *pB42AD-WRKY* construct was co-transformed with *pLaczi-W-box1*, *pLaczi-W-box2*, and *pLaczi* empty vector (negative control) into the yeast strain EGY48, respectively. The positive binding was indicated by the blue color of the yeast cells grown on SD/-Trp-Ura+Gal+Raf+X-Gal media. As shown from the Y1H assays, the defense response related WRKY8 and senescence related WRKY22, WRKY28, and WRKY70 had the ability to identify and bind to the W-box1 motif (Figure 10). Among them, WRKY8 and WRKY28 also had a weak binding to the W-box2 element (Figure 10). Moreover, WRKY15 was able to bind to two W-box elements and the *pLaczi* empty vector, indicative of the presence of self-activation. In addition, W-box motif nearby the start codon was bound with a higher frequency, suggesting that this WRKY binding site might be more important for the regulation of *AtWAKL10* expression. Overall, these results further reinforce the evidence that AtWAKL10 is involved in SA-related stress responses, especially pathogen defense and leaf senescence.

### 2.10. A Brief Analysis of Potential Transcriptional Changes Specifically Mediated by AtWAKL10 during Natural Leaf Senescence

**Figure 11:**
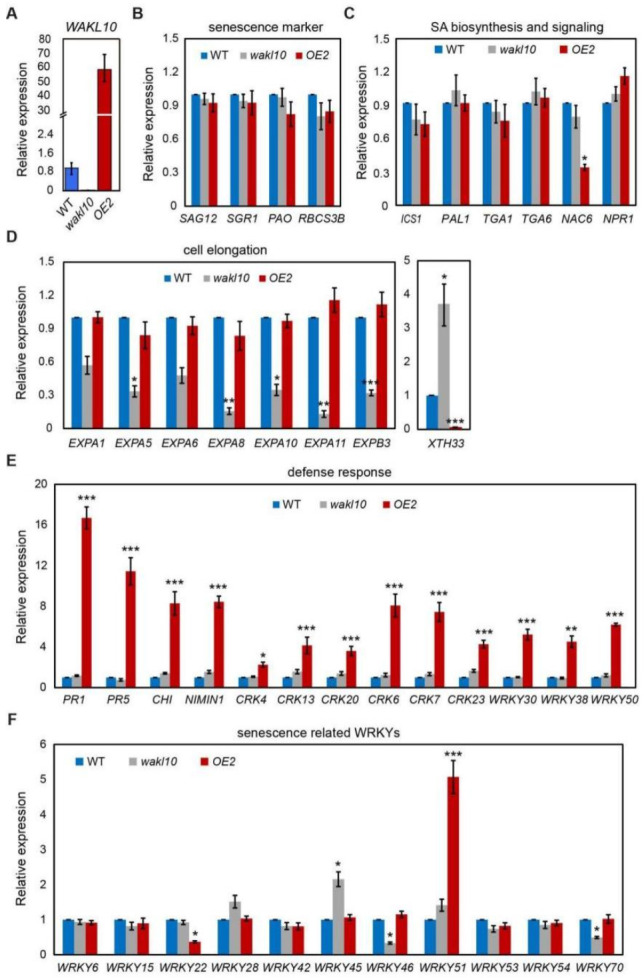
Identification of specific transcriptional changes in *atwakl10* mutant and *AtWAKL10* overexpression plants with the same degree of senescence as wild type. For all three genotypes as indicated, the sixth and seventh leaves with 20% leaf area from leaf tip yellowing were collected. The relative transcript levels of *AtWAKL10* (**A**), senescence markers (**B**), genes involved in SA synthesis and signaling (**C**), cell expansins (**D**), defense responsive genes (**E**), and senescence related WRKY TFs (**F**) were quantified. Data are presented as means ± SE (*n* = 30), and significant differences compared with the wild type (Col-0) were determined by Student’s *t*-test (* *p* < 0.05; ** *p* < 0.01 and *** *p* < 0.001).

Previously, numerous studies have described the common transcriptional changes during leaf senescence. To exclude general developmental responses and explore specific functions of AtWAKL10 under natural leaf senescence, senescent leaves 6 and 7 with the similar degree of yellowing (less than 20% leaf area from leaf tip yellowing) were collected from 7-week-old *atwakl10* mutant, *AtWAKL10 OE2* line, and wild type plants. Based on its uncovered and predicted functions, transcriptional changes of key genes involved in hormone biosynthesis and signaling, defense response, cell elongation, as well as those encoding senescence related WRKYs and WAK/WAKL members were determined using specific primers (Figure 11; Supplementary Figure S4 and Table S2).

To start, the lack and overexpression of AtWAKL10 was further confirmed in senescing leaves from *atwakl10* mutant and *AtWAKL10 OE2* line, respectively (Figure 11A). Transcript abundance of four senescence marker genes including *SAG12*, *SGR1*, *PAO*, and *RBCS3B* in mutant and *AtWAKL10 OE2* line were quantified, which were similar to that in wild type, suggesting that these genotypes were at a comparable level of senescence (Figure 11B). Then, we measured the expression levels of a range of genes functioning in biosynthesis and signaling of SA, JA, ABA, and ethylene (Figure 11B; Supplementary Figure S4). In general, expression of these genes in both mutant and *AtWAKL10 OE2* line did not differ from that in wild type except for a few scattered changes. Specifically, the expression of NAC transcription factor NAC DOMAIN CONTAINING PROTEIN 6 (NAC6)/ANAC092/ORE1, a key positive regulator of age-dependent senescence, was significantly down-regulated in *AtWAKL10 OE2* line. Moreover, in both mutant and overexpression plants, a series of jasmonate ZIM-domain (JAZ) transcriptional suppressors in the JA signaling pathway were down-regulated to varying degrees, especially for *JAZ1, JAZ7* and *JAZ8* in *AtWAKL10 OE2* line, which were ~30 times lower than that in wild type (Supplementary Figure S4A). In addition, the bZIP transcription factor ABSCISIC ACID INSENSITIVE5 (ABI5), a core component of ABA signaling, was greatly upregulated in *atwakl10* mutant (Supplementary Figure S4B). Since these transcription factors have been found to play a crucial role in promoting crosstalk among ABA, JA, SA, and ethylene in regulating different biological processes [48], the regulation of leaf senescence by AtWAKL10 should be tightly related to hormone pathways. Given the dismal transcriptional changes in hormone pathways, the underlying regulatory mechanism may be related to protein interaction or protein stability, which needed to be explored in future studies.

The most transcriptional changes mediated by AtWAKL10 occurred in genes encoding proteins involved in controlling cell expansion and defense response (Figure 11D,E; Supplementary Figure S5). The former included the *EXPANSIN A1* (*EXPA1*), *EXPA5, EXPA6, EXPA8, EXPA10, EXPA11*, and *EXPB3*, all of which were specifically inhibited in *atwakl10* mutant (Figure 11D). Oppositely, only the overexpression lines had significantly elevated transcript levels of defense responsive genes. Part of these genes were related to innate immune response including *PATHOGENESIS-RELATED GENE 1 (PR1*), *PR5*, *NIM1-INTERACTING 1* (*NIMIN-1*), and *CHITINASE* (*CHI*), some were associated with programmed cell death, e.g., *CYSTEINE-RICH RECEPTOR-LIKE PROTEIN KINASE 4* (*CRK4*), *CRK13*, and *CRK20*, others for response to SA, such as *WRKY30* and *WRKY38*, and the remaining were involved in response to fungus and bacterium including *CRK6*, *CRK7*, *CRK23*, and *WRKY50* (Figure 11E; Supplementary Figure S5). Notably, one gene named *xyloglucan endotransglycosylase/hydrolase 33* (*XTH33*), which plays a critical role in cell wall extension and modification [49,50], was significantly upregulated and downregulated in *atwakl10* mutant and in the overexpression lines, respectively (Figure 11D; Supplementary Figure S5). It further indicated that the intrinsic role of AtWAKL10 as a regulator of cell wall elongation contributed to its regulation of leaf senescence. These transcriptional changes in both mutant and overexpression lines were only observed during senescence but not at the non-senescence stage (Supplementary Table S3), suggesting that AtWAKL10 was required for maintaining cell elongation and cell wall structure of senescing leaves and was able to mediate defense response upon aging (Figure 11E).

WRKYs have been known to constitute a large proportion of *SAGs*, and it is an interesting question whether their expression levels are specifically affected by the deletion or overexpression of AtWAKL10 during leaf senescence (Figure 11F). It could be seen that transcript levels of most senescence associated WRKYs were the same as those of wild type. However, in the *atwakl10* mutant, genes encoding two negative regulators WRKY46 and WRKY70 [51] and one positive regulator WRKY45 [52] were significantly downregulated and upregulated, respectively, which was highly consistent with its early senescence phenotype. On the other hand, the high expression of *AtWAKL10* led to significant down-regulation of *WRKY22*, which was identified as a positive regulator of dark-induced senescence [53]. Moreover, similar to other SA-responsive WRKYs, *WRKY51* was also significantly up-regulated in the *AtWAKL10* overexpression line. 

In addition, relative expression levels of previously characterized *WAKs* (*WAK1-3*) and *WAKLs* (*WAKL1-7*) were also determined (Supplementary Figure S4). As was shown, overexpression of AtWAKL10 was accompanied by enhanced transcription of *WAK1* and *WAK3*, while its deletion resulted in an approximately 7-fold up-regulation of *WAKL5*. These results provided a possibility that AtWAKL10 may be co-expressed with *WAK1* and *WAK3*, and functionally redundant with WAKL5 during leaf senescence, which required further confirmation at the protein level or through functional genetics such as mutant crosses and complementation or suppression tests. 

## 3. Discussion

For the response to unexpected stresses and changing environmental challenges, plants have evolved special mechanisms, such as cell wall sensing systems, to relay external signals to intracellular effectors to trigger defense responses and minimize potential damage. Receptor-like kinases are indispensable sensors that contribute to intercellular communication, cellular signaling and plant-pathogen interactions [31,32,33]. Most of the well characterized RLKs belong to the leucine-rich repeat RLKs (LRR-RLKs) and are mainly involved in tissue and organ development, hormone perception, and stress response [33,54,55]; only a few RLKs are identified to participate in the regulation of leaf senescence [56,57,58,59,60]. Since an appropriate delay of senescence can improve crop yield potential and agronomic traits due to prolonged photosynthesis, exploring more senescence related RLKs and underlying mechanisms can provide practical theoretical guidance for agricultural production. In this study, we found that *AtWAKL10*, encoding a member of the cell wall-associated class of RLKs, was highly up-regulated in senescing leaves (Figure 1 and Figure 2). Deletion of AtWAKL10 exacerbated age-dependent and dark-induced leaf senescence, with the loss-of-function mutant having higher expression of senescence marker genes and lower chlorophyll content compared with wild type during natural leaf senescence (Figure 3 and Figure 4). In parallel, plants overexpressing AtWAKL10 exhibited the oppositely delayed senescence phenotype, which further confirmed that AtWAKL10 exerted negative control over leaf senescence.

Our results showed that GFP signals from *35S::GFP* were cytosolic while signals from *35S::AtWAKL10-GFP* were mostly in membrane systems. A prediction through the UniProt program (https://www.uniprot.org/, accessed on 19 April 2021) also shows that AtWAKL10 is localized in the plasma membrane. Moreover, other members of its family such as WAKL4 [61] and WAKL6 [62] in Arabidopsis as well as its potential homologs in *Brassica napus* [63] were reported to be present on cell membranes. Further analyses are needed to confirm the subcellular localization of AtWAKL10.

WAKs and WAKLs are a special class of RLKs that are divided into four groups based on the sequence and structure similarities in Arabidopsis [35]. AtWAKL10 is one of the six members of Group III with WAKL9, WAKL11, WAKL13, WAKL17, and WAKL18, which have similar structure features to the Group II members (WAKL1-6 and WAKL22). In addition to the protein sequence, some stress- and growth-associated motifs identified in the *AtWAKL10* promoter were also present in other WAK members [36,62], suggesting that these members may be involved in similar biological processes and probably have similar transcriptional regulatory control. Typically, a positive role of AtWAKL10 in regulating early defense responses had also been confirmed for WAK group members, with some being greatly up-regulated by fungal and bacterial pathogens, SAR inducing conditions, and defense signaling molecules SA and MeJA [38,39,40,41]. In fact, the case seems to be more complicated for AtWAKL10, which is required to respond to intricate conditions such as convergence of different pathogens and microbes [41], and here, multiple hormones involved in leaf senescence (Figure 8 and Figure 9). Since the interrelated transcription of *AtWAKL10* and genes encoding other PM localized receptor kinases was likely to promote the recognition and transduction of external adverse signals and activation of cellular signaling [41,64,65], identifying AtWAKL10-associated proteins and the key transcription factors or cascades for coordinately regulating hormone signals is necessary.

Transcriptomic analyses of leaves of different developmental stages and those under darkness stress have identified numerous WRKY TFs differentially expressed during leaf senescence [16,66,67]. These WRKYs play distinct regulatory roles in leaf senescence. For example, WRKY54 and WRKY70 act as negative regulators, whereas WRKY53 is identified as an accelerator of the onset of senescence [51,68,69]. *WRKY22*, a target gene of WRKY53, is induced by darkness stress, while WRKY22, similar to WRKY6, exerts positive control over dark-induced senescence [53,70]. WRKY28 contributes to SA biosynthesis and positively regulates age- and light-mediated leaf senescence [71,72,73]. In fact, most senescence-regulating WRKYs are involved in SA-related biological processes, which further support the large overlapping of transcriptomic changes and signaling pathways between SA-mediated stresses and age-dependent leaf senescence [11,74]. Similarly, *AtWAKL10* can be induced by pathogen infection and its promoter is rich in WRKY TFBS and SA responsive elements (Figure 7), strengthening the possibility that AtWAKL10 is involved in the regulation of leaf senescence. Previously, several WRKY TFs have been predicted as transcription regulators of *AtWAKL10*, including WRKY2/3/4/6/10/14/18/26/33/40/56/61/65/71 [36,37], and WRKY28 and WRKY15 were highly co-expressed with *AtWAKL10* [35]. These findings are partially in accordance with our results, especially for the WRKY28, exhibiting a strong binding to the W-box1 nearby the start codon (Figure 10). WRKY15 may be a candidate but it showed a weak binding and can be self-activated. WRKY70, a well-known TFs involved in defense and stress response [75,76,77,78], is bound to the W-box1 on the *AtWAKL10* promoter. Since these multifunctional WRKYs are recruited to assist a variety of biological processes, their coordinate regulation of *AtWAKL10* places it as a key node that integrates and responds to signals of different levels [70,72,79,80,81,82]. It could not be ignored that these WRKYs play different regulatory roles in leaf senescence, and some factors such as WRKY54 did not affect its transcriptional expression, but may exert controls by physical interactions, which makes the control of leaf senescence by AtWAKL10 more complicated. In addition, given the limited study scope, more senescence-related WRKYs such as WRKY33 [83], WRKY45 [52], WRKY42 [84], and WRKY75 [85] should be validated.

As previously reported, *AtWAKL10* was mainly induced by SA-related stimuli [41]. Here, we also showed that it could be significantly activated by multiple hormones, though to varying degrees (Figure 8). Surprisingly, although there was no JA responsive element but four SA responsive elements in the promoter of *AtWAKL10*, it was maximally induced by ABA and JA but not SA. This was probably attributed to the concentration of the inducer. For example, SA functions in a concentration-dependent manner in which it promotes growth and development at low concentration but acts as a stress inducer at high concentration [86,87]. In addition, unlike the continuous up-regulation of *AtWAKL10* under ABA and JA treatments, SA was inclined to trigger short-term and periodic response, which was consistent with previous studies [43].

Leaf senescence is a degenerative process with large-scale degradation of organelles and structural components, leading to the destruction of the cell wall. Therefore, maintaining leaf cell elongation and cell wall structure is an effective means to prevent senescence. It was commonly accepted that WAKs were crucial for cell elongation, while decreased levels of WAKs caused dose-dependent inhibition of cell elongation and the resulting smaller rosette leaves [36,37]. However, there is so far no experimental evidence that AtWAKL10 is involved in regulating these processes. Cell elongation is controlled by different classes of wall proteins, among which expansins are mostly characterized factors that play crucial roles in organ growth, cell elongation, cell wall structure, and extensibility [88,89,90]. Here, deletion and overexpression of AtWAKL10 did not cause obvious alterations in the size and number of rosette leaves, organ morphology, bolting time, and expression of expansins compared with the wild type during the non-senescence developmental stages [43] (Supplementary Table S3). However, a significant down-regulation of expansins was specifically observed in the *atwakl10* mutant upon leaf senescence, indicating that AtWAKL10 may be required for the expression of expansins, and such interconnection seemed to be age-dependent. Since the expression of expansins was not changed because of the upregulation of *AtWAKL10*, they could not be co-expressed with *AtWAKL10*. Unlike the WAKs and group II WAKL members, which could mediate common cell growth and organ development, the regulation of cell elongation and extension by AtWAKL10 was more like a stress responsive mechanism. Specifically, AtWAKL10 has been reported to mediate responses to pathogen infection and drought stress [43]. Both processes involve disruption of cell walls that was caused by invasion of external pathogens and altered cell tension, respectively. Expansins, as core regulators of cell wall extension, play crucial roles in resisting these stress conditions [91,92,93]. Still notably, there was a significant increase in expression of *ABI5* in *atwakl10* mutant during leaf senescence. As a core ABA signaling component, *ABI5* is always upregulated to cope with drought stress [94,95,96,97]. Thereby, our results may provide a potential mechanism for the elevated resistance of the *atwakl10* mutant to drought stress [43]. 

Another significant senescence-specific change was the consistent up-regulation of defense response genes in *AtWAKL10 OE* lines. Most of these genes such as *PR1*, *PR5*, *CHI*, and *WRKY30*/*38*/*50*/*51* positively respond to both exogenous SA treatment and pathogen-induced endogenous SA biosynthesis, indicating that SA signaling is involved in AtWAKL10 mediated senescence regulatory mechanisms. However, it was interesting that genes encoding key enzymes for SA biosynthesis, e.g., *Isochorismate Synthase 1 (ICS1)* [98] and *PHE AMMONIA LYASE 1* (*PAL1*), and core components for SA reception and signal transduction such as *NONEXPRESSER OF PR GENES 1* (*NPR1*) [99], *TGACG SEQUENCE-SPECIFIC BINDING PROTEIN 1* (*TGA1*), and *TGA6* [100], were not differentially expressed compared with wild type. Since accumulation of SA can promote the transition from growth to senescence, it was possible that overexpression of AtWAKL10 triggered the downstream defense response, which acted as a feedback signal to inhibit excessive accumulation and signal transduction of SA. Notably, *NAC6*/*ORE1*/*ANAC092*, as the signal transduction junction of SA and ethylene to promote leaf senescence [26,101], was significantly down-regulated by elevated accumulation of AtWAKL10. Similarly, WRKY70 could bind to the promoter of *AtWAKL10* (Figure 7), but the lack of AtWAKL10 during leaf senescence led to obvious down-regulation of *WRKY70*, which can generally be activated by SA [77]. The specific regulatory mechanism for these changes remains to be further studied.

## 4. Materials and Methods

### 4.1. Plant Materials and Growth Conditions

In this study, *Arabidopsis thaliana* Columbia ecotype (Col-0) was used as the wild type. The homozygous *atwakl10* mutant (SALK_132887; http://abrc.osu.edu/, accessed on 20 August 2020) was obtained from the Arabidopsis Biological Resource Center (ABRC) and genotyped by PCR using the AtWAKL10-LP and AtWAKL10-RP primers (Supplementary Table S1), with the precise T-DNA insertion site determined by Sanger sequencing. Both overexpression lines (*OE1* and *OE2*) were generated in the Col-0 background and confirmed by quantitative real-time PCR.

Seeds of all genotypes were surface sterilized with 70% (*v/v*) ethanol for 5 min and stratified for 3 days before being transferred to growth chambers with 22 °C/22 °C (day/night), continuous light and 120 μmol m^−2^ s^−1^ in all experiments. For soil-based growth, seeds were sown on a soil, vermiculite, and perlite mixture (3:1:1) in a randomized design, with the senescence progress being monitored (YL, young leaves, 3-week-old plants; NS, non-senescence plants with fully expanded leaves; ES, early senescence from the initiation of leaf tip yellowing to the yellowing leaf area < 25%; LS, late senescent stage, with more than 40% leaf area yellowing). For hormone-induced expression pattern analysis, seedlings were cultured in 1/2 MS medium (Sigma Aldrich, St. Louis, MO, USA) for 10 days before being transferred into 1/2 MS medium supplemented with 50 μM ABA (Sigma Aldrich), 100 μM JA (Sigma Aldrich), 100 μM SA (Solarbio, Beijing, China), and 10 μM ETH (Solarbio), respectively. Samples were collected at 1 h, 3 h, and 6 h, frozen in liquid nitrogen, and stored at −80 °C for RNA extraction. For hormone-induced senescence response, leaf discs with equal area (diameter = 1 cm) from the leaves 7, 8, and 9 of 4- and 6-week-old plants were treated with or without 10 μM ABA, 100 μM JA, and 100 μM SA, respectively. Images were taken at 0 d, 2 d, and 5 d of treatments as indicated. For all measurements, three biological replicates were carried out.

### 4.2. Developmental Expression and Promoter Analysis

The developmental tissue expression pattern of *AtWAKL10* was analyzed through Arabidposis eFB Brower (http://bar.utoronto.ca, accessed on 8 November 2020). Furtherly, the expression levels for *AtWAKL10* in *A. thaliana* wild type plants at different stages of plant development were obtained from the developmental data set in GENEVESTIGATOR v3 (https://genevestigator.com, accessed on 20 December 2020).

To identify potential growth and stress responsive elements as well as transcription factors binding sites, the 2000 bp region upstream of *AtWAKL10* CDS was analyzed via the PlantCARE program (http://bioinformatics.psb.ugent.be/webtools/plantcare/html/, accessed on 23 December 2020).

### 4.3. RNA Extraction and Quantitative RT-PCR

Total RNAs from treated and untreated tissues were extracted using Ultrapure RNA Kit with TRIzol according to manufacturer’s instructions (cwbiotech, Beijing, China). Reverse transcriptions were performed using the HiScript III All-in-one RT SuperMix Perfect for qPCR (R333, Vazyme, Nanjing, China) and quantitative RT-PCR was performed using ChamQ Universal SYBR qPCR Master Mix (Q711-02, Vazyme, Nanjing, China) and on a 7500 Fast Real-Time PCR System (Applied Biosystems, Waltham, MA, USA) with biological triplicates. *UBC21* and *ACT2* were used as the reference genes and other primers used here were listed in Supplementary Table S1. Data analyses were carried out based on the 2^−ΔΔCt^ method [102].

### 4.4. Generation of AtWAKL10 Overexpression Lines (OEs) and AtWAKL10::GUS Lines

For generation of *AtWAKL10* (AT1G79680) overexpression lines, the corresponding coding sequence (CDS) was amplified by PCR using the AtWAKL10-CDS-F and AtWAKL10-CDS-R primers (Supplementary Table S1). The purified PCR product was ligated into enzyme digested pRI101 with NdeI and EcoRI by Infusion (Clontech, Bejing, China). Similarly, for generation of *AtWAKL10::GUS* lines, a fragment about 2.3 kb upstream of the start codon was cloned using the primer pair AtWAKL10-Pro-F and AtWAKL10-Pro-R, integrated into pBI101 at the BamHI and SalI digestion sites by Infusion (Clontech, Bejing, China). The correct constructs were transformed into *Agrobacterium* competent cells (GV3101), followed by transformation of *A. thaliana* (Col-0) by *Agrobacterium*-mediated floral dip [103]. The positive transgenic plants were screened on 1/2 MS medium supplemented with 40 mg/L kanamycin (Sigma Aldrich), and homozygous T3 lines were used for further study.

### 4.5. Subcellular Localization

The coding sequence of AtWAKL10 excluding the stop codon was amplified and cloned into the pCHF3-cGFP vector under the control of the cauliflower mosaic virus (CaMV) 35S promoter using pEASY^®^-Basic Seamless Cloning and Assembly Kit according to the manufacturer’s instructions (TransGen Biotech, Beijing, China). *Agrobacterium tumefaciens* strain (GV3101) carrying *35S::AtWAKL10-GFP* or *35S::GFP* (empty pCHF3 vector as a control) construct was infiltrated into the leaf epidermis of 4-week-old *N. benthamiana* as previously described [104]. Proper expression accumulation for GFP fusion protein usually takes 3 d, with the GFP signals observed and imaged using a confocal laser microscope (TCS-SP8 Leica, Wetzlar, Germany).

### 4.6. Yeast One-Hybrid Analysis

Yeast one-hybrid assays were performed according to the EGY48 Yeast One-Hybrid System (Clontech, Bejing, China). The full-length CDS sequences of various WRKY transcription factors were firstly cloned into pEasy-Blunt vector (Transgen Biotech, Beijing, China), then recombined into the EcoRI digested pB42AD (Clontech, Bejing, China), respectively. Two ~200 bp individual fragments harboring a W-box motif from the promoter of *AtWAKL10* were cloned into SalI digested pLacZi-2u (Clontech, Bejing, China), respectively. A mixture of combinations of plasmids, carrier DNA, and PEG/LiAc together with the yeast EGY48 strain (Clontech, Bejing, China) was cultured in a water bath at 30 °C for 30 min, followed by a water bath heat shock at 42 °C for 15 min. The supernatant was discarded after centrifugation at 5000 rpm for 40 s, and competent cells were resuspended with ddH_2_O, and placed on SD/-Trp-Ura mediums (Coolaber, Bejing, China) for growth at 30 °C for 4 d. Large colonies were grown on SD/-Trp-Ura+Gal+Raf+X-Gal (20 mg/mL, Coolaber, Bejing, China) medium at 29 °C for 4 d. The positive binding between protein and DNA motif was identified based on the blue color of colonies.

### 4.7. Measurement of Chlorophyll Content, Fv/Fm and Ion Leakage

The extraction of total chlorophyll from 30~50 mg leaves was performed using 100% methanol by rotating in the dark at 4 °C until all the chlorophyll was removed. Chlorophyll content was measured at 666 and 653 nm with a spectrophotometer (ClarioSTAR, BMG LABTECH, Offenburg, Germany) and calculated as previously described [105]. For determination of chlorophyll fluorescence Fv/Fm, the whole plant or individual leaves were exposed to 120 µmol m^−2^ s^−1^ of actinic light provided by IMAGING-PAM M-series Chlorophyll Fluorescence System (LI-6400-40 LCF, Walz, Effeltrich, Germany) after a 15~20 min of dark acclimation [106]. For ion leakage measurement, leaves were placed in double-distilled water and shaken at room temperature. The initial conductivity was measured after 1 h using a digital conductivity meter (Thermo Fisher Scientific Traceable, Fisher Scientific, Hampton, NH, USA). Then, samples were boiled for 15 min, cooled down to room temperature and the final conductivity was measured. Ion leakage is calculated as a percentage of the initial conductivity to the final conductivity.

### 4.8. Dark-Induced Senescence

All plants were grown on soil and randomly mixed in growth chambers at 22 °C under continuous light. Two independent assays related to dark-induced senescence were performed on attached leaves and detached leaves, respectively, as described previously [107]. For the assay based on attached leaves, leaves 4, 5, and 6 from uniformly developed 25-day-old wild type, *atwakl10* mutant, and *AtWAKL10* overexpression lines were covered with aluminum foil wrap for 7 d. For darkness stress on detached leaves, five true leaves (3, 4, 5, 6, and 7) from 25-day-old plants of each genotype were excised and placed onto moistened filter paper inside foil-wrapped Petri dishes. Images were taken at 0 d, 3 d, and 5 d, respectively. 

### 4.9. Statistical Analysis

All data analyses in this study were performed based on at least three biological replicates. Statistically significant differences were determined using Student’s *t*-test (* *p* < 0.05, ** *p* < 0.01, and *** *p* < 0.001). Value in each graph gives the mean value ± SE of replicates. 

## 5. Conclusions

In this study, we functionally characterized the Arabidopsis cell wall associated receptor-like kinase AtWAKL10, which exhibited the highest expression levels in naturally senescing leaves. Expression of *AtWAKL10* could also be induced by exogenous ABA, JA, and SA, while its promoter could be bound by a number of defense and senescence associated WRKY transcription factors. Overexpression of AtWAKL10 effectively delayed the aging-dependent and stress-induced leaf senescence while the loss-of-function *atwakl10* mutant showed precocious senescence. The inhibited and accelerated natural leaf senescence phenotypes were probably attributed to up-regulated expression of defense responsive genes and down-regulation of cell expansins, respectively. In conclusion, the receptor-like kinase AtWAKL10 negatively regulates leaf senescence, which may be related to its intrinsic role in regulating cell elongation and defense response, in which hormone signaling may act as an integration bridge.

## Data Availability

Data is contained within the article or Supplementary Materials.

## Supplementary Materials

Supplementary materials are available online at https://www.mdpi.com/article/10.3390/ijms22094885/s1. Figure S1. The predicted developmental tissue expression pattern of *AtWAKL10* generated from GENEVESTIGATOR. Figure S2. Identification of homozygous *atwakl10* mutants and determination of the T-DNA insertion site. Figure S3. AtWAKL10 negatively regulated hormone mediated senescence in Arabidopsis. Figure S4. Analysis of specific transcript changes caused by mutation or overexpression of AtWAKL10 during natural leaf senescence. Figure S5. Further confirmation of the relative expression levels of genes encoding cell expansins and those involved in defense responses in senescent leaves of *AtWAKL10 OE1* line. Table S1. List of primers used in Y1H assays, genotyping and generation of transgenic lines. Table S2. List of primers used in qRT-PCR transcript analysis. Table S3. List of genes quantified in *atwakl10* mutant and *AtWAKL10 OE2* line in senescent (S) and non-senescent (NS) leaves.
