# Supplementary material for: AtWAKL10, a Cell Wall Associated Receptor-Like Kinase, Negatively Regulates Leaf Senescence in Arabidopsis thaliana
Source: Int J Mol Sci. 2021 May 5;22(9):4885. doi: 10.3390/ijms22094885 (PMC8124439; doi:10.3390/ijms22094885)
Supplement: Supplementary file 1 [file ijms-22-04885-s001.zip › ijms-1186353-supplementary/Supplementary Figure S1.pdf]

Supplementary Figure S1

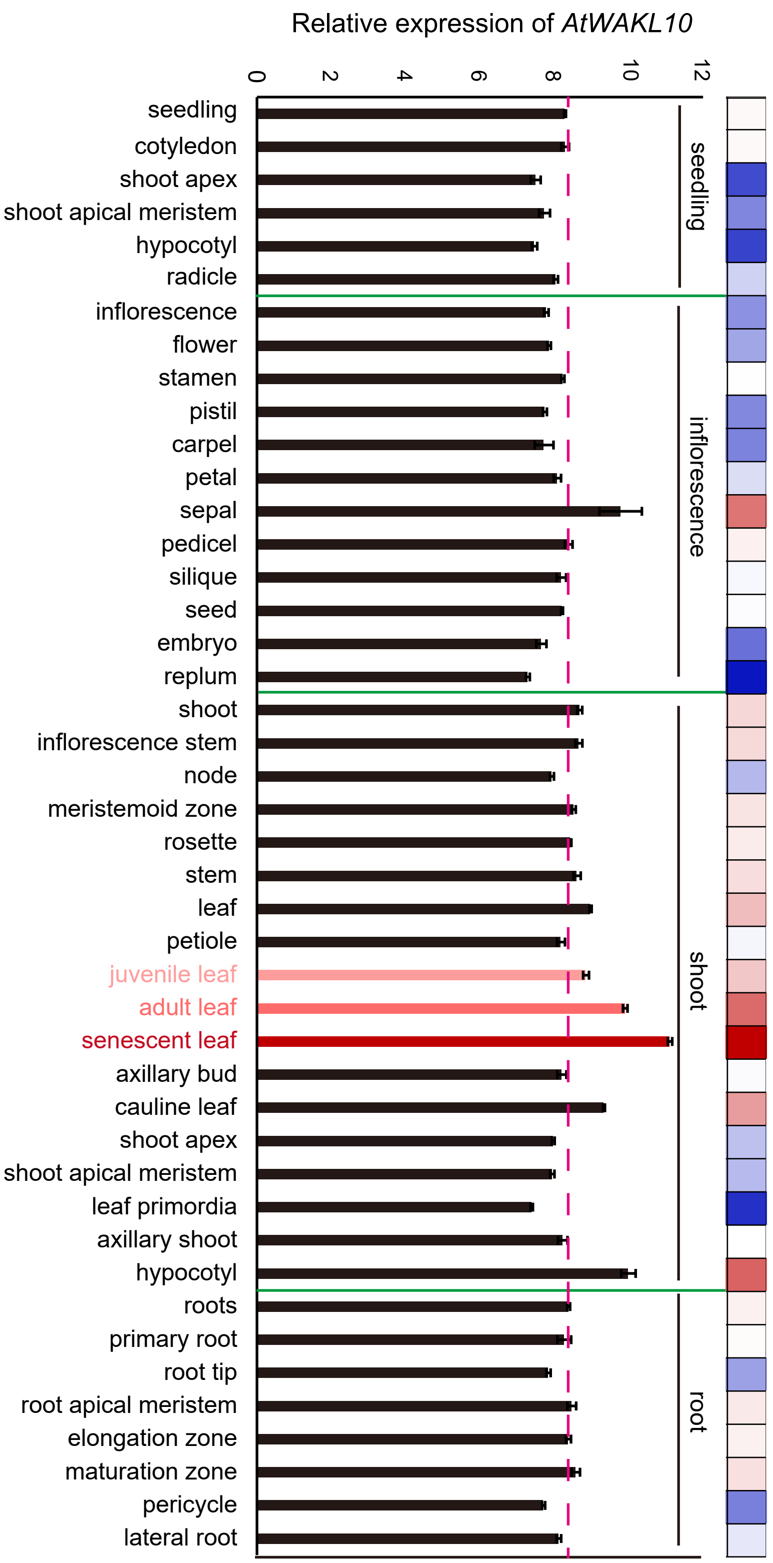

Supplementary Figure S1. The predicted developmental tissueexpression pattern of *AtWAKL10* generated from GENEVESTIGATOR.
