# Supplementary material for: AtWAKL10, a Cell Wall Associated Receptor-Like Kinase, Negatively Regulates Leaf Senescence in Arabidopsis thaliana
Source: Int J Mol Sci. 2021 May 5;22(9):4885. doi: 10.3390/ijms22094885 (PMC8124439; doi:10.3390/ijms22094885)
Supplement: Supplementary file 1 [file ijms-22-04885-s001.zip › ijms-1186353-supplementary/Supplementary Figure S2.pdf]

Supplementary Figure S2

A

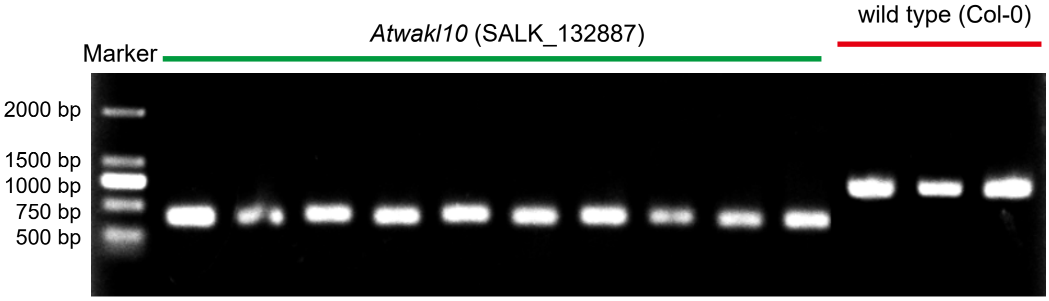

B

```
                                    LP
AtWAKL10     CCATAAATCACCTTGTACACCGTATATAATATCGTACACCTTCACCTATTGTGTACACCTCATATTCAAAAGATAAAACC  80
SALK_132887  --------------------------------------------------------------------------------  0

AtWAKL10     ATTTTTGTCAAGTGATAATATACAAAACAAAGTTTAAATAATAATAATATGAGATTAGTGTACATAAAAGTGTACAAATG  160
SALK_132887  --TTTTGCCGG--------ATTCGGAACCACCATCAAA------------------------------------------  28

AtWAKL10     TTAGTGTACATAAGTAAAAATGTATAATCATTACTTTCATTTAATGGTATACACCATTATGGTTAATTTAATAGTATACA  240
SALK_132887  ----------------------------CAGGATTTTCGCCTGCTGGGGCAAACCAGCGTGG------------------  62

AtWAKL10     TGATGGTGTACACCATTATATTTTTATTAATTTATGAACAATTATTATTTTATTTTATAAATATGAGATAAATAGAAATA  520
SALK_132887  --------------------------------------------------------------------------------  62

AtWAKL10     TTTTAGAAGATATGTATAAAATGATTTGGTGTATAAGTGTTAGTGTACAAAACTATGTTTTAATGCTGGTGTACGAGAAT  400
SALK_132887  --------------------ACCGCTTGCTGCA-----------------------------------------------  75

AtWAKL10     TATAATTAAAAACATATAAATCATCTTGTTTTTGGAAGAAGAAATTATTTTTTAAAAATAATTTATTTTTATAAAATTCA  480
SALK_132887  --------------------------------------------------------------------------------  75

AtWAKL10     AAAAGATGAATATGAAAAAAAAAATAACTATAATATAATAAAAATAGTTCTAAATAGTTAAATAATATAAAAAGTTTGAC  560
SALK_132887  --------------------------------------------------------------------------------  75

AtWAKL10     CCATATTTTTTCATATAAAGTAAAAGGTAAAATGGTATTTGACATATTTTTTGTCTTTTAGCTTATTACACATAACCTCT  640
SALK_132887  ----ACTCTCTC-----------AGGGCCAGGCGGTG-------------------------------------------  97

AtWAKL10     ATTTCACAAAAAAAGTAAAAAAAGTGTCTATTTCACAAATTTCTAAAAAAACTACCTACTTCTCAAATTACTTTTTAAAA  720
SALK_132887  --------------------------------------------------------------------------------  97

AtWAKL10     AGTGTCTACTATTCTAATAAGCCTAAAACAATAAGCTGAGCTAGATTCACTTCTCTATGTTTCTTGCGTTCATGCGTGTG  800
SALK_132887  -----------------------AAGGGCAATCAGCTG------------TTGCCCGTCTCACTGGTG------------  130

AtWAKL10     ACCTTACAAAAGAAAATCCAAAAAACGCAACTCTGACTAATGTTTCAGCAGATCATAACACATAATAAACATGCATGAGC  880
SALK_132887  -------AAAAGAAAAACC--------------------------------ACCCCAGTACATTAAAAACGTCCGCAA--  169

AtWAKL10     TCTACAGATACTTGTTCTCTTTTTGATATTTAATTAGCCACGTTTTAATCTAGAGTCGGATTGTGATAGTTACAGTGGAA  960
SALK_132887  ---------------------TGTGTTATTAAGTTGTCTAAGCGTCAATTT-----------------------------  199

AtWAKL10     ATTTCTCCTCGCGACCTAAACCAGTCACGTTTTGATATTTTTCTTTTTTCTCGTAAATAAATGGAGGAATATAAAATATT  1040
SALK_132887  -------------GTTTACACC------------------------------------------------ACAATATATC  218

                                   ↓ insertion site
AtWAKL10     GAAATTCAAACAAGGAGGCTCACTGAAAGCAAGAAAAGCTTTCACTAGGAGGACTAGAGAAACAAAGACTGACCAGAGAA  1120
SALK_132887  ----------------------CTGAAAGCAAGAAAAGCTTTCACTAGGAGGACTAGAGAAACAAAGACTGACCAGAGAA  276

AtWAKL10     GAAAATATGAAAGCGAAATTCAAAGATGAAGACTTATGATTCATTGCTGGGTCAAACTAAACACAAGATTTTTCCTTCGC  1200
SALK_132887  GAAAATATGAAAGCGAAATTCAAAGATGAAGACTTATGATTCATTGCTGGGTCAAACTAAACACAAGATTTTTCCTTCGC  356

AtWAKL10     TCATCCTTACAAAACCCGTCTCTGCTTTTAGCTTTTCTTGAGCAGATCATAAACAGAAAACATTAATATGAGCTCTAATT  2280
SALK_132887  TCATCCTTACAAAACCCGTCTCTGCTTTTAGCTTT---------------------------------------------  391
                                                         RP                   *** start codon

AtWAKL10     GTAGTTGTTCTCTT 1294
SALK_132887  -------------- 391
```

Supplementary Figure S2. Screening of homozygous *atwakl10* mutants and determination of the T-DNA insertion site. Homozygous mutant plants were confirmed by PCR-based genotyping with the LP (left primer) and RP (right primer). The PCR product was sent for sequencing. Sequence alignment was carried out using the Sequence Manipulation Suite.
