# Supplementary material for: AtWAKL10, a Cell Wall Associated Receptor-Like Kinase, Negatively Regulates Leaf Senescence in Arabidopsis thaliana
Source: Int J Mol Sci. 2021 May 5;22(9):4885. doi: 10.3390/ijms22094885 (PMC8124439; doi:10.3390/ijms22094885)
Supplement: Supplementary file 1 [file ijms-22-04885-s001.zip › ijms-1186353-supplementary/Supplementary Figure S3.pdf]

Supplementary Figure S3

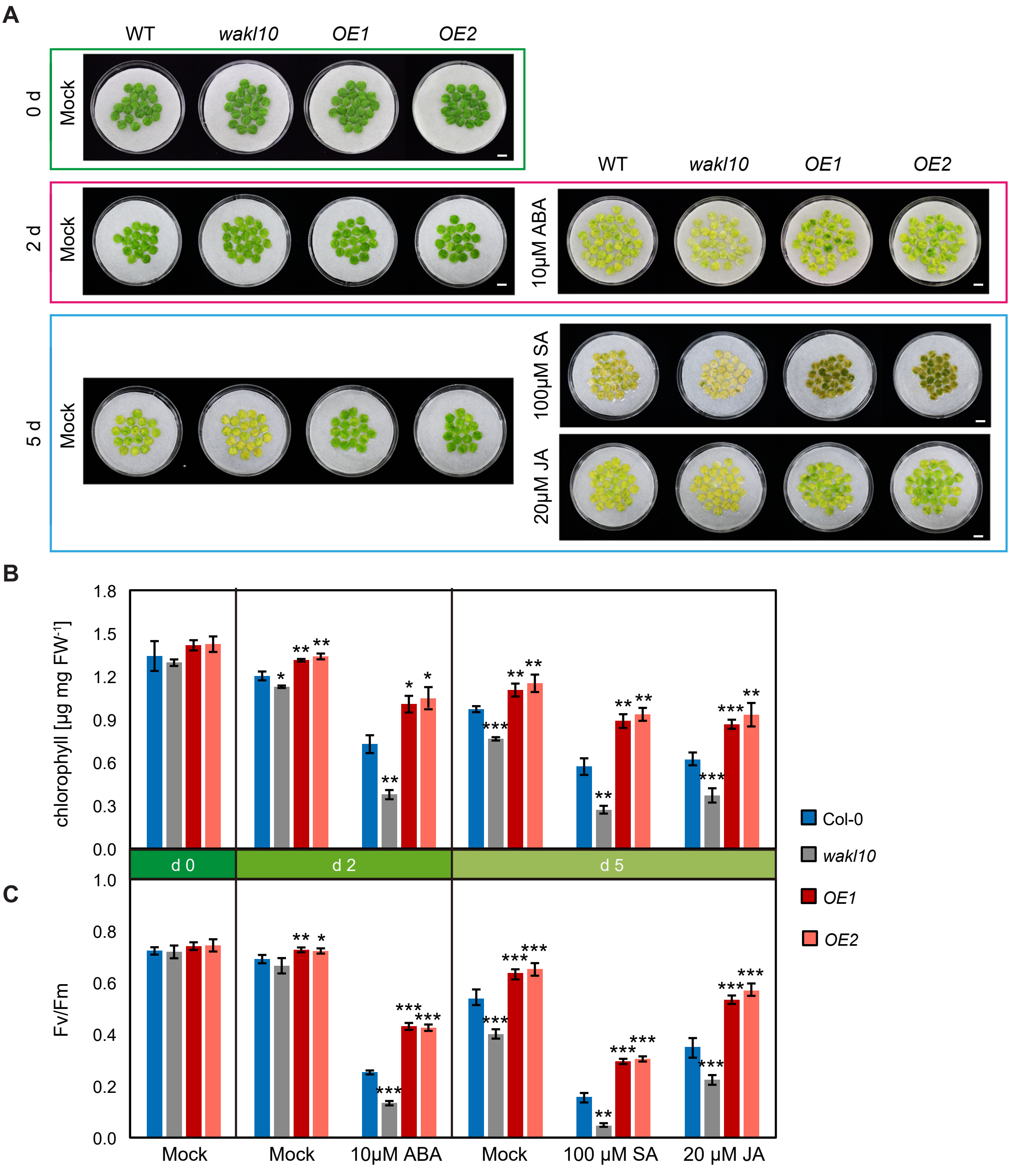

Supplementary Figure S3. AtWAKL10 negatively regulated hormone mediated senescence in Arabidopsis. (A) Hormone responsive phenotypes of leaf discs with equal area (diameter = 1 cm) taken from leaves 7, and 8 of 6-week-old wild type, *atwakl10* mutant and *AtWAKL10 OE* plants. Leaf discs were treated and untreated (Mock) with various hormones as indicated. Representative images of leaf discs with obvious senescence phenotypes were taken after 0 d of mock treatment (green box), 2 d of mock and 10 μM ABA treatment (light blue box), 5 d of mock, 100 μM SA and 20 μM JA treatments (purple box), respectively. Mock treated leaf discs were used to monitor natural senescence progression. (B) The chlorophyll concentrations and (C) Fv/Fm in hormone treated and untreated leaf discs as indicated in (A). Data were presented as means ± SE (n >20). For all conditions, statistically significant differences compared with the wild type at each time point were determined by Student's *t-test* (**p* < 0.05; ***p* < 0.01 and ****p* < 0.001).
