# Supplementary material for: AtWAKL10, a Cell Wall Associated Receptor-Like Kinase, Negatively Regulates Leaf Senescence in Arabidopsis thaliana
Source: Int J Mol Sci. 2021 May 5;22(9):4885. doi: 10.3390/ijms22094885 (PMC8124439; doi:10.3390/ijms22094885)
Supplement: Supplementary file 1 [file ijms-22-04885-s001.zip › ijms-1186353-supplementary/Supplementary Figure S4.pdf]

Supplementary Figure S4

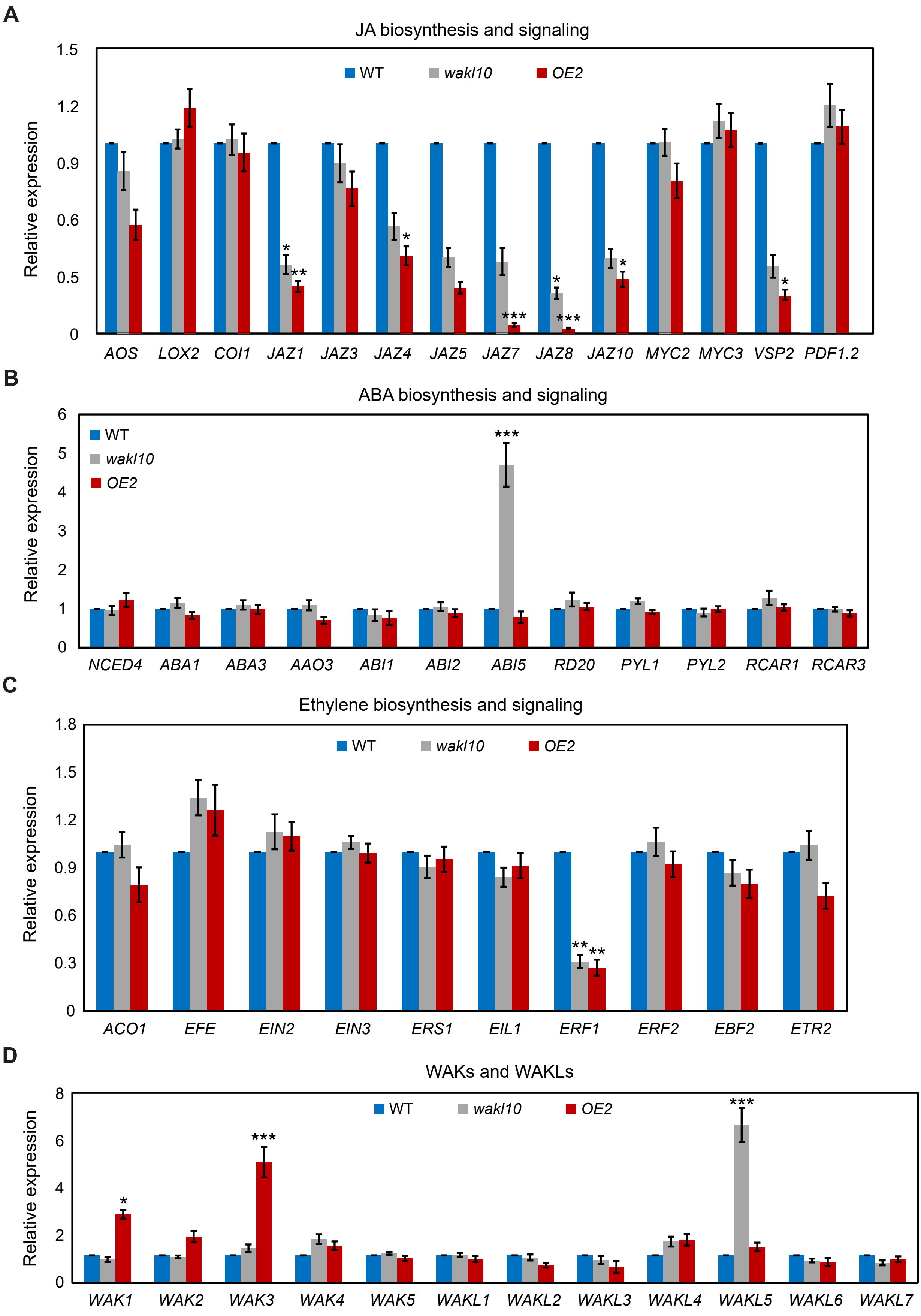

Supplementary Figure S4. Analysis of specific transcript changes caused by mutation or overexpression of AtWAKL10 during natural leaf senescence. For all three genotypes, rosette leaves 6 and 7 with 20% leaf area from leaf tip yellowing were collected. The relative transcript levels of genes encoding key enzymes and components of JA, ABA and ethylene signaling as well as members of WAKs and WAKLs were determined. Data were presented as means ± SE of biological troplicates. Significant differences for *wakl10* mutant and *OE2* line compared with the wild type (Col-0) were determined by Student's *t-test* (**p* < 0.05; ***p* < 0.01 and ****p* < 0.001).
