# Supplementary material for: AtWAKL10, a Cell Wall Associated Receptor-Like Kinase, Negatively Regulates Leaf Senescence in Arabidopsis thaliana
Source: Int J Mol Sci. 2021 May 5;22(9):4885. doi: 10.3390/ijms22094885 (PMC8124439; doi:10.3390/ijms22094885)
Supplement: Supplementary file 1 [file ijms-22-04885-s001.zip › ijms-1186353-supplementary/Supplementary Figure S5.pdf]

Supplementary Figure S5

	Gene ID	Gene Name	*wakl10* vs WT	*OE1* vs WT	*OE2* vs WT	
	AT1G79680	WAKL10	0.03	115.64	61.50	
senescence markers	AT5G45890	SAG12	0.96	0.95	0.93	
senescence markers	AT4G22920	SGR1	0.94	0.92	0.89	
senescence markers	AT3G44880	PAO	0.98	0.94	0.92	
senescence markers	AT5G38410	RBCS3B	0.84	0.88	0.85	
cell expansins	AT1G69530	EXPA1	0.57	1.23	1.00	
cell expansins	AT3G29030	EXPA5	0.34	0.96	0.84	
cell expansins	AT2G28950	EXPA6	0.48	1.12	0.93	
cell expansins	AT2G40610	EXPA8	0.16	0.98	0.84	
cell expansins	AT1G26770	EXPA10	0.35	1.13	0.97	
cell expansins	AT1G20190	EXPA11	0.13	1.32	1.16	
cell expansins	AT4G28250	EXPB3	0.32	1.05	1.12	
cell expansins	AT1G10550	XTH33	3.72	0.02	0.03	***
defense responsive genes	AT2G14610	PR1	1.17	19.67	16.69	***
defense responsive genes	AT1G75040	PR5	0.76	16.64	11.45	***
defense responsive genes	AT2G43570	CHI	1.41	12.48	8.28	***
defense responsive genes	AT1G02450	NIMIN1	1.54	14.34	8.44	***
defense responsive genes	AT3G45860	CRK4	1.07	2.87	2.25	*
defense responsive genes	AT4G23210	CRK13	1.57	7.34	4.15	***
defense responsive genes	AT4G23280	CRK20	1.39	4.12	3.61	**
defense responsive genes	AT4G23140	CRK6	1.24	11.34	8.08	***
defense responsive genes	AT4G23150	CRK7	1.33	10.75	7.44	***
defense responsive genes	AT4G23310	CRK23	1.65	5.16	4.28	***
defense responsive genes	AT5G24110	WRKY30	1.03	4.85	5.22	***
defense responsive genes	AT5G22570	WRKY38	0.94	3.44	4.52	**
defense responsive genes	AT5G26170	WRKY50	1.30	3.56	2.32	*
defense responsive genes	AT5G64810	WRKY51	1.41	9.23	6.19	***
defense responsive genes	AT2G46400	WRKY46	0.33	1.05	1.15	
defense responsive genes	AT3G56400	WRKY70	0.49	0.98	1.02	

Supplementary Figure S5. Further confirmation of the relative expression levels of genes encoding cell expansins and those involved in defense responses in senescent leaves of *AtWAKL10 OE1* line. Leaves 6 and 7 with less than 20% leaf area yellowing were collected from wild type, *wakl10* mutant and both *AtWAKL10 OE* lines for transcriptional expression analysis. The relative expression levels of tested transcripts in *AtWAKL10 OE1* line showed a similar trend as that in *AtWAKL10 OE2* line. Data were averages of biological triplicates. Significant differences for *OE1* compared with the wild type were indicated by asterisks and determined by Student's *t-test* (*$p < 0.05$; **$p < 0.01$ and ***$p < 0.001$).
